# Phase I Trial of Ipatasertib Plus Carboplatin, Carboplatin/Paclitaxel, or Capecitabine and Atezolizumab in Metastatic Triple-Negative Breast Cancer

**DOI:** 10.1093/oncolo/oyad026

**Published:** 2023-04-06

**Authors:** Yuan Yuan, Susan E Yost, Yujie Cui, Christopher Ruel, Mireya Murga, Aileen Tang, Norma Martinez, Daniel Schmolze, James Waisman, Niki Patel, Lalit Vora, Lusine Tumyan, Mari Bozoghlanian, Daphne Stewart, Paul H Frankel

**Affiliations:** Department of Medical Oncology & Therapeutics Research, City of Hope Comprehensive Cancer Center, Duarte, CA, USA; Division of Medical Oncology, Cedars-Sinai Cancer, Los Angeles, CA, USA; Department of Medical Oncology & Therapeutics Research, City of Hope Comprehensive Cancer Center, Duarte, CA, USA; Department of Statistics, City of Hope Comprehensive Cancer Center, Duarte, CA, USA; Department of Statistics, City of Hope Comprehensive Cancer Center, Duarte, CA, USA; Department of Medical Oncology & Therapeutics Research, City of Hope Comprehensive Cancer Center, Duarte, CA, USA; Department of Medical Oncology & Therapeutics Research, City of Hope Comprehensive Cancer Center, Duarte, CA, USA; Department of Medical Oncology & Therapeutics Research, City of Hope Comprehensive Cancer Center, Duarte, CA, USA; Department of Pathology, City of Hope Comprehensive Cancer Center, Duarte, CA, USA; Department of Medical Oncology & Therapeutics Research, City of Hope Comprehensive Cancer Center, Duarte, CA, USA; Department of Medical Oncology & Therapeutics Research, City of Hope Comprehensive Cancer Center, Duarte, CA, USA; Department of Radiology, City of Hope Comprehensive Cancer Center, Duarte, CA, USA; Department of Radiology, City of Hope Comprehensive Cancer Center, Duarte, CA, USA; Department of Radiology, City of Hope Comprehensive Cancer Center, Duarte, CA, USA; Department of Medical Oncology & Therapeutics Research, City of Hope Comprehensive Cancer Center, Duarte, CA, USA; Department of Statistics, City of Hope Comprehensive Cancer Center, Duarte, CA, USA

**Keywords:** Ipatasertib, platinum, atezolizumab, triple–negative breast cancer

## Abstract

**Background:**

This trial evaluated the safety and efficacy of ipatasertib in combination with carboplatin, carboplatin/paclitaxel, or capecitabine/atezolizumab in patients with metastatic triple–negative breast cancer (mTNBC).

**Methods:**

Eligibility criteria were mTNBC, RECIST 1.1 measurable disease, no prior use of platinum for metastatic disease (Arms A and B), and no prior exposure to immune checkpoint inhibitor (Arm C). Primary endpoints were safety and RP2D. Secondary endpoints were progression–free survival (PFS), response rate, and overall survival.

**Results:**

RP2D for Arm A (*n* = 10) was ipatasertib 300 mg daily, carboplatin AUC2, and paclitaxel 80 mg m^−2^ days 1, 8, and 15 every 28 days. RP2D for Arm B (*n* = 12) was ipatasertib 400 mg daily and carboplatin AUC2 days 1, 8, and 15 every 28 days. RP2D for Arm C (*n* = 6) was likely ipatasertib 300 mg 21 days on 7 days off, capecitabine 750 mg m^−2^, twice a day, 7 days on 7 days off, and atezolizumab 840 mg days 1 and 15 every 28 days. The most common (≥10%) grade 3-4 AEs at RP2D for Arm A (*N* = 7 at RP2D) were neutropenia (29%), diarrhea (14%), oral mucositis (14%), and neuropathy (14%); Arm B had diarrhea (17%) and lymphopenia (25%); and Arm C had anemia, fatigue, cognitive disturbance, and maculopapular rash (17% each). Overall responses at RP2D were 29% Arm A, 25% Arm B, and 33% Arm C. PFS was 4.8, 3.9, and 8.2 months for patients on Arms A, B, and C, respectively.

**Conclusions:**

Continuous dosing of ipatasertib with chemotherapy was safe and well-tolerated. Further study is warranted in understanding the role of AKT inhibition in treatment of TNBCs.

**Trial registration:**

NCT03853707.

## Teaser

More effective therapeutic strategies targeting underlying genomic drivers, as well as novel combinations, are urgently needed for treatment of triple-negative breast cancer (TNBC). This article evaluates the safety and efficacy of ipatasertib in combination with carboplatin, carboplatin/paclitaxel, or capecitabine/atezolizumab in patients with metastatic TNBC.

**Implications for Practice**

Ipatasertib is a selective pan-AKT inhibitor and displays synergy with chemotherapy preclinically. This study was designed to determine the dose, safety, and preliminary efficacy of ipatasertib plus carboplatin, carboplatin/paclitaxel, or capecitabine/atezolizumab in mTNBC. The combination was safe and well-tolerated at the recommended doses, with empirically higher efficacy seen in tumors harboring PIK3CA/AKT/PTEN alterations. In addition, androgen receptor-positivity was associated with an empirically higher response rate. This study was stopped early due to a Phase III study combining ipatasertib plus paclitaxel that failed to show PFS or OS benefit. Further analysis is warranted in understanding the role of AKT inhibition in the treatment of TNBCs.

## Introduction

Metastatic triple-negative breast cancer (mTNBC), defined as lack of estrogen receptor (ER), progression receptor (PR), and human epidermal growth factor receptor 2 (HER2) overexpression, remains a disease of unmet need. This is largely attributed to tumor heterogeneity and chemotherapy resistance.^1^ A number of novel agents have recently been granted FDA approval, including PARP inhibitors for germline BRCA mutated tumors,^2,3^ immune checkpoint inhibitor (ICI) for programmed death-ligand 1 (PD-L1) positive TNBC,^4-6^ and antibody drug conjugates targeting the Trop-2 receptor.^7,8^ Despite these advances, the median overall survival (OS) of patients with mTNBC remains limited at approximately 18 months.^9^ In the ASCENT trial, the median OS was 12.1 months (95% CI, 10.7-14.0) with sacituzumab govitecan as 3+ lines of therapy, and 6.7 months (95% CI, 5.8-7.7) with chemotherapy (hazard ratio 0.48; *P* < .001).^8^ Therefore, more effective therapeutic strategies targeting underlying genomic drivers, as well as novel combinations are urgently needed.

The Cancer Genome Atlas data demonstrated that the most frequently altered genomic drivers in TNBC include the phosphatidylinositol 3-kinase-AKT-mTOR (PI3K-AKT-mTOR) signaling pathway.^10,11^ The mTOR inhibitor everolimus in combination with eribulin or carboplatin has shown moderate activity.^12,13^ Ipatasertib is a selective ATP–competitive small–molecule inhibitor of AKT that preferentially targets active phosphorylated Akt (pAkt) and is potent in cell lines with evidence of Akt activation. Ipatasertib displays synergy when combined with taxanes or other chemotherapeutic agents (gemcitabine, platinum, 5-FU, doxorubicin, and paclitaxel) in vitro.^9,12-14^ In the LOTUS trial, combination of paclitaxel 80 mg m^−2^ (days 1, 8, and 15) and ipatasertib 400 mg po days 1-21 every 28 days showed modestly improved progression–free survival (PFS). More pronounced improvement was seen in tumors with alteration of the PIK3CA/AKT/PTEN pathway.^15^ The combination regimen was well-tolerated with 23% of grade ≥3 diarrhea and 18% grade 3 neutropenia. Phase III IPATunity trial failed to confirm the efficacy of adding ipatasertib to paclitaxel in both TNBC (Cohort A)^16^ and ER+ MBC as first–line therapy (Cohort B),^17^ which is intriguing. This controversial result may indicate a different chemotherapy backbone is needed for optimized therapeutic effect.

DNA–damaging agents such as platinum drugs are active in TNBC. The randomized Triple Negative Breast Cancer Trial (TNT) showed no overall response rate (ORR) difference between carboplatin and docetaxel in the overall population: 31.4% vs 34% (*P* = .66).^18^ However, carboplatin showed higher ORR and longer PFS in comparison with docetaxel in patients with gBRCA^+^ mTNBC: ORR 68% vs 33% (*P* = .01) and PFS 3.1 months (95% CI: 2.4, 4.2) vs 4.4 months (95% CI: 4.1, 5.1).^19^ A high proportion of TNBC tumors exhibit BRCAness, which indicate these tumors are highly sensitive to platinum agents.^20,21^ A weekly carboplatin–paclitaxel regimen has been tested in multiple clinical trials and has shown efficacy and acceptable safety profiles.^22^ Weekly paclitaxel (100 mg m^−2^) was combined with weekly carboplatin AUC 2, days 1, 8, and 15 on a 4–week schedule in a cohort of advanced breast cancer patients. The regimen was effective with a response rate (RR) of 62% and was well tolerated.^23^ We previously found that in the patient–derived xenograft model of mTNBC, carboplatin and ipatasertib were synergistic in tumor suppression.^24^ Based on this evidence, we hypothesize that carboplatin or carboplatin/paclitaxel in combination with ipatasertib would have a synergistic effect in mTNBC. Hence Arms A and B of this clinical trial were designed to determine the dose and safety of ipatasertib plus carboplatin or carboplatin/paclitaxel to obtain initial evidence of efficacy. A continuous dosing schedule of ipatasertib was used in both Arm A and Arm B.

The Impassion130 study demonstrated the addition of atezolizumab (anti–PD-L1) to *nab*-paclitaxel improved PFS and OS outcomes in PD-L1 positive mTNBC in the first–line setting.^4^ Despite disappointing results from Impassion131, some patients did gain benefit from the ipatasertib-paclitaxel combination.^25^ However, it remains to be seen whether non-taxane chemotherapy plus ICI will be beneficial in mTNBC. The CREATE-X study showed benefit of adjuvant capecitabine for patients with residual disease after standard neoadjuvant chemotherapy.^26^ The combination of capecitabine and ICI pembrolizumab was shown to be safe and well tolerated in 2 clinical trials.^27,28^ AKT inhibitors have shown immune modulatory effects in the preclinical setting. Mittendorf et al. showed that MDA-MB-468 cells treated with the AKT inhibitor MK-2206 resulted in significantly decreased PD-L1 expression, further linking the PI3K/AKT/PTEN pathway signal to PD-L1 regulation.^29^ In a cohort of patients receiving MK-2206 therapy preoperatively, a favorable immune profile including increased CD8+ cells and increased expression of interferon genes were observed.^30^ These data support the rationale of combining an AKT inhibitor with immunotherapy. In a Phase IB study, the combination of ipatasertib, atezolizumab, and paclitaxel or *nab*-paclitaxel showed a promising RR of 73% in patients (*N* = 26) with mTNBCs. Interestingly, responses were high irrespective of PD-L1 status or PI3K/AKT/PTEN alteration status.^31^ Based on this evidence, the aim of Arm C was to test the safety and preliminary efficacy of the triplet combination of ipatasertib (intermittent dosing), capecitabine, and atezolizumab in patients with mTNBC.

## Materials and Methods

This open-label single institutional Phase I/Ib trial was conducted between May 2019 and June 2022 with institutional review board (IRB 18496) approval at the City of Hope National Cancer Center. The trial was conducted in accordance with the World Medical Association Declaration of Helsinki, International Conference on Harmonization Good Clinical Practice guidelines, and the US code of federal regulations. Informed consent forms were signed by all patients prior to study entry. This study is registered at the ClinicalTrials.gov under number NCT03853707.

### Eligibility Criteria

Main eligibility criteria were: ≥18 years; histologically confirmed TNBC defined by ER or PR ≤ 10% by IHC and HER2 negative per ASCO/CAP guidelines; ECOG performance status 0-1; life expectancy ≥3 months; RECIST 1.1 measurable disease for Arm C only (patients on Arms A and B could have non–measurable disease), available baseline archival tissue for PIK3CA/AKT/mTOR status, and adequate organ function. Main exclusion criteria included unresolved grade 3 toxicities, prior exposure to PIK3CA/AKT/mTOR pathway inhibitors, prior exposure to carboplatin (Arms A and B) for metastatic disease, prior exposure to paclitaxel (Arm A) for metastatic disease, prior exposure to capecitabine or ICIs (Arm C) in the metastatic setting, or untreated or unstable brain metastasis or leptomeningeal metastasis.

### Study Endpoints and Assessments

The primary objective of the study was to evaluate safety and determine the recommended Phase II dose (RP2D) of ipatasertib plus carboplatin and paclitaxel (Arm A), carboplatin (Arm B), or capecitabine and atezolizumab (Arm C). Secondary endpoints were RR, PFS, and OS. Responses were assessed by RECIST 1.1, and safety analysis was carried out based on toxicities assessed by CTCAE 5.0. Immune-related adverse events (irAEs) were also collected for Arm C.

Patients underwent tumor assessments with CT scan of chest, abdomen, pelvis, and bone scan of at baseline, every 3 cycles (12 weeks) following treatment initiation regardless of dose delays, until radiographic disease progression per RECIST v1.1 or intolerance. All measurable and evaluable lesions were re-assessed at each subsequent tumor evaluation. An objective response was confirmed by repeat assessments ≥4 weeks after initial response.

### Statistical Design for Arms A and B

The combination of paclitaxel and ipatasertib was determined to be safe and well tolerated in previous Phase Ib (PAM 4983g)^32^ and Phase II LOTUS studies as: ipatasertib 400 mg po daily 3 weeks on/1 week off and paclitaxel 80 mg m^−2^ days 1, 8, and 15 of every 4-week cycle.^15,33^

**Figure 1.**
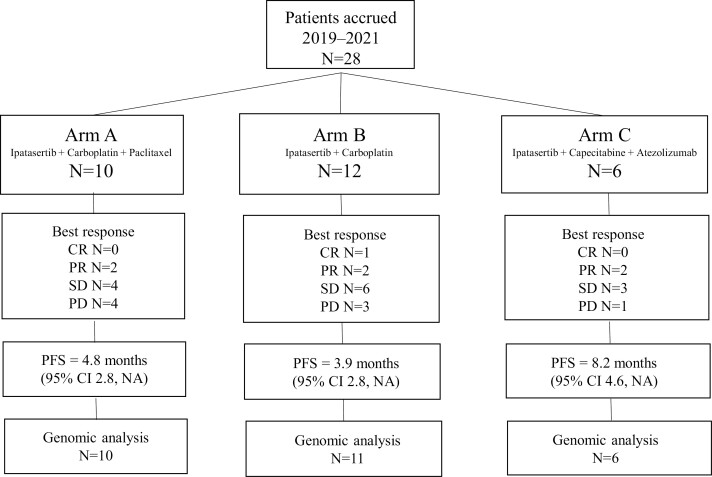
Treatment Arms showing patients included in the study (*n* = 28) grouped by Arm A (Ipatasertib + Carboplatin + Paclitaxel, *n* = 10), Arm B (Ipatasertib + Carboplatin, *n* = 12), and Arm C (Ipatasertib + Capecitabine + Atezolizumab, *n* = 6). Best response and PFS are indicated for each arm. Number of patients with samples for correlative analyses including genomic sequencing (*n* = 27) is indicated (1 patient in arm B did not have sufficient sample for genomic analysis). CR, complete response; PR, partial response; SD, stable disease; PD, progression of disease; PFS, progression–free survival.

Treatment Arms are shown in Fig. 1. For Arm A, ipatasertib was administered at a starting dose level of 400 mg oral daily for 28 days with carboplatin AUC 2 paclitaxel at 80 mg m^−2^ IV on days 1, 8, and 15 of every 28 days. For Arm B, ipatasertib was administered at a starting dose level of 400 mg oral daily for 28 days plus carboplatin AUC 2 on days 1, 8, and 15 every 28 days. For Arm C, ipatasertib was administered at a starting dose level of 300 mg oral daily for 21 days on and 7 days off, capecitabine at a dose of 750 mg m^−2^ 7 days on and 7 days off, and atezolizumab at a dose of 840 mg IV on days 1 and 15 every 28 days. For Arms A and B, continuous dosing of ipatasertib was used. For Arm C, ipatasertib was used for 21 days on and 7 days off. For the Phase I/Ib design, the Phase I Queue (IQ) 3 + 3 design was used.^34^ This method has been shown to reduce study duration by an average of approximately 23% for a typical Phase I study, with a median increase in the number of patients of 1-4, while reducing the number of patients turned away due to lack of slots.

To confirm the RP2D obtained from the dose escalation, and for an initial assessment of response, correlatives, and PFS estimates, an additional cohort of patients was enrolled until the number treated and evaluable for DLT considerations at the recommended Phase II dose was 14 patients for Arm B and Arm A. All eligible patients who started treatment at the recommended Phase II dose were considered in the calculation of the RR.

Arms A and B had an expected sample size of 14 patients, for an expected total sample-size of 28 patients. Protocol allowed for the addition of cohorts (6-8 patients) if level 1 was not well-tolerated (a lower dose was necessary).

For Arm C safety-lead in, a 3-at-risk design was utilized to assess toxicity for the combination therapy. The DLT period was 1-cycle (28 days). Each participant remained on the dosing level according to the escalation dose level they were enrolled in, and intra-dose level escalations were not allowed, even if the MTD was defined at a higher dose level. If a patient came off study in the first 28 days for any reason outside of toxicity (unrelated AE, withdrawal of consent, progression of disease, etc), this patient was not considered as evaluable for DLT and this patient was replaced.

When a maximum tolerable dose (MTD) level was defined by the dose escalation portion of the study, and the recommended Phase II dose (RP2D not to exceed the MTD) was selected, additional patients were accrued to confirm the tolerability of the regimen. The plan was for at least 12 patients to be treated at the RP2D to confirm tolerability. Additional patients beyond the 12 at the RP2D could be accrued if the total number of patients accrued did not exceed 21 patients. With 12 patients, any specific severe toxicity with 20% incidence would be observed with 93% probability.

Survival endpoints were evaluated using Kaplan-Meier methods. Clinical activity was described based on the secondary objectives, with a description of the activity based on PD-L1 status. Other biological correlative studies were considered exploratory in the context of this limited Phase I study.

### Tumor Immune Biomarker

Tumor biopsies were formalin-fixed paraffin-embedded (FFPE). Percentage of stromal tumor infiltrating lymphocytes (TILs) in tumor was evaluated using H&E diagnostic sections per International Immuno-Oncology Biomarker Working Group on Breast Cancer Guidelines.^35^ Genomic sequencing was performed using Tempus xT version 4 assay consisting of 648 genes with single nucleotide variants, indel, and translocations measured by next-generation sequencing.^36^ The copy number gain limit of detection was set as 30% and copy number loss at 40% tumor purity. Tumor mutational burden was reported as the quantity of somatic single nucleotide variations (SNVs) and indels (including benign) measured as mutations per million coding base pairs. PD-L1 was reported through the commercial testing platform Tempus using SP142 antibody. PD-L1 positive was defined by at least ≥1% on immune cells.

## Results

### Patients

A total of 28 patients with metastatic TNBC were enrolled between May 2019 and April 2021: 10, 12, and 6, respectively, to Arms A, B, and C (Fig. 1). The study was stopped early due to sponsor’s withdrawal of support responding to negative Phase III IPATunity trial in mTNBC.^16^ Baseline patient characteristics are listed in Table 1. Median age was 56 years (33-77); 43% were non–Hispanic White, 36% Hispanic, 14% were African American, and 7% Asian. A total of 26 (92%) had infiltrating ductal carcinoma, 1 (4%) had infiltrating lobular carcinoma, and 1 (4%) was metaplastic. Median prior lines of therapy for metastatic disease was 0 (range 0-2), including 16 patients (57%) with no prior lines of therapy for mTNBC, 9 (32%) with 1 prior line, and 3 (11%) with 2 prior lines. Major sites of metastasis were: 15 non–visceral, 4 liver, and 9 lung/pleura/diaphragm.

**Table 1.** Baseline patient characteristics (*N* = 28).

Characteristic	*N* (%)^a^
Median age (range)	56 (33-77)
Race/ethnicity	
Non–Hispanic White	12 (43%)
Hispanic	10 (36%)
African American	4 (14%)
Asian	2 (7%)
Histology at initial diagnosis	
IDC	26 (92%)
ILC	1 (4%)
Metaplastic	1 (4%)
Tumor stage at initial diagnosis	
Stage I	7 (25%)
Stage II	7 (25%)
Stage III	12 (43%)
Stage IV	2 (7%)
Grade at initial diagnosis	
1	1 (4%)
2	6 (21%)
3	19 (68%)
Unknown	2 (7%)
AR status at time of metastasis^b^	
Positive	9 (32%)
Negative	18 (64%)
Not done	1 (4%)
Prior surgery	23 (82%)
Prior radiation	19 (68%)
Prior lines for metastatic disease	
0	16 (57%)
1	9 (32%)
2	3 (11%)
Site of metastasis	
Non–visceral	15 (54%)
Liver	4 (14%)
Lung	9 (32%)
BRCA status	
BRCA1/2 positive	4 (14%)
BRCA1/2 negative	23 (82%)
BRCA1/2 not done	1 (4%)
ECOG status	
0	18 (64%)
1	10 (36%)

^a^Median (range).

^b^AR positive, IHC AR ≥ 2+ and ≥30%; *n* (%).

### Toxicities

For Arm A, 3 patients developed DLT (1 grade 3 diarrhea, 1 grade 3 stomach pain, and 1 persistent grade 2 diarrhea) leading to dose delay (less than 75% planned ipatasertib dosing) (Table 2). Due to DLTs, ipatasertib dose level was de-escalated from 400 mg to 300 mg daily. An additional 7 patients were treated in Arm A with the RP2D dose: ipatasertib 300 mg daily, carboplatin AUC2, paclitaxel 80 mg m^−2^, days 1, 8, and 15, every 28 days. Grade 3 AEs at RP2D were: 2/7 (29%) leukopenia, 2/7 (29%) lymphopenia, 2/7 (29%) neutropenia, and 1/7 (14%) each of diarrhea, oral mucositis, and peripheral sensory neuropathy. One patient in Arm A had both grade 3 oral mucositis and grade 3 peripheral sensory neuropathy (Table 3). Of a total of 12 patients treated in Arm B, grade 4 toxicity included 1/12 (8%) lymphopenia; and grade 3 toxicities included 2/12 (17%) diarrhea, 2/12 (17%) lymphopenia, and 1/12 (8%) each anemia, hyperglycemia, and maculopapular rash. One patient in Arm B had both grade 3 diarrhea and grade 3 hyperglycemia (Table 3). Arm C grade 3 toxicities included 1/6 (17%) each anemia, fatigue, cognitive disturbance, and maculopapular rash. One patient in Arm C had both grade 3 fatigue and grade 3 cognitive disturbance. One patient had grade 3 maculopapular rash attributed to atezolizumab (Table 3).

**Table 2.** DLTs within the first cycle (representing 5 unique patients).

Arm	AE	Grade
Arm A	Diarrhea	3
Arm A	Stomach pain	3
Arm A	Diarrhea^a^	2
Arm B	Rash maculopapular	3
Arm C	Rash maculopapular^b^	3

^a^Due to persistent grade 2 diarrhea, patient had dose delay, less than 75% planned ipatasertib dose.

^b^irAE attributed to atezolizumab.

**Table 3.** Adverse events at RP2D per CTCAE 5.0 (*N* = 25).

Treatment arm	Adverse event	Grade 2	Grade 3	Grade 4
Arm A (Carbo/Taxol + Ipat), *n* = 7 (excluding 3 patients at 400 mg)	All arm A events	7 (100%)	5 (71%)	0 (0%)
	Diarrhea	5 (71%)	1 (14%)	
	Leukopenia	3 (43%)	2 (29%)	
	Lymphopenia	1 (14%)	2 (29%)	
	Neutropenia	4 (57%)	2 (29%)	
	Anemia	4 (57%)		
	Mucositis, oral		1 (14%)^a^	
	Peripheral sensory neuropathy	3 (43%)	1 (14%)^a^	
	Nausea/vomiting	1 (14%)		
	Thrombocytopenia	1 (14%)		
	Hyperglycemia	2 (29%)		
	Anorexia	1 (14%)		
Arm B (Carbo + Ipat), *n* = 12	All Arm B events	11 (92%)	5 (42%)	1 (8%)
	Diarrhea	5 (42%)	2 (17%)^b^	
	Lymphopenia	2 (17%)	2 (17%)	1 (8%)
	Leukopenia	5 (42%)		
	Anemia	5 (42%)	1 (8%)	
	Hyperglycemia	1 (8%)	1 (8%)^b^	
	Dry mouth	1 (8%)		
	Dyspepsia	1 (8%)		
	Fatigue	2 (17%)		
	Fever	1 (8%)		
	Flushing	1 (8%)		
	Gastroesophageal reflux disease	1 (8%)		
	Hypertension	1 (8%)		
	Hypoalbuminemia	1 (8%)		
	Hypomagnesemia	1 (8%)		
	Hypophosphatemia	1 (8%)		
	Mucositis, oral	1 (8%)		
	Nausea/vomiting	1 (8%)		
	Neutropenia	6 (50%)		
	Pain	1 (8%)		
	Thrombocytopenia	2 (17%)		
	Restlessness	1 (8%)		
	Nail infection	1 (8%)		
	Rash, maculopapular		1 (8%)	
Arm C (Cape +Ipat + Atezo), *n* = 6	All Arm C events	4 (67%)	3 (50%)	0 (0%)
	Anemia	1 (17%)	1 (17%)	
	Anorexia	1 (17%)		
	Arthralgia	1 (17%)		
	Diarrhea	1 (17%)		
	Fatigue	1 (17%)	1 (17%)^c^	
	Fever	1 (17%)		
	Lymphopenia	2 (33%)		
	Leukopenia	1 (17%)		
	Neutropenia	2 (33%)		
	Malaise	1 (17%)		
	Pain	1 (17%)		
	Pain in extremity	1 (17%)		
	Cognitive disturbance		1 (17%)^c^	
	Rash, maculopapular		1 (17%)	

^a^One patient had both grade 3 oral mucositis and grade 3 peripheral sensory neuropathy.

^b^One patient had both grade 3 diarrhea and grade 3 hyperglycemia.

^c^One patient had both grade 3 fatigue and grade 3 cognitive disturbance.

As previously mentioned, of the 10 patients in Arm A, 3 had DLTs including 2 patients with grade 2-3 diarrhea and 1 patient with grade 3 stomach pain within the first 28-day cycle, which led to de–escalation to dose -1 with ipatasertib (300 mg daily). Of the 12 patients in Arm B, 1 had grade 3 maculopapular rash in cycle 1, which led to dose delay of ipatasertib (<75% completion) during the first 28 days. Of the 6 patients in Arm C, 1 had grade 3 maculopapular rash attributed to atezolizumab. After appropriate treatment, both patients with maculopapular rash had resolution of rash (Table 2).

Median (range) of cycles completed was 4 (2-15), including 4 (2-7) for Arm A, 4 (2-15) for Arm B, and 6 (2-10) for Arm C. A total of 11 (39%) had dose delay, including 4 (40%) in Arm A, 6 (50%) in Arm B, and 1 (17%) in Arm C. Ten (36%) had dose reduction, including 6 (60%) in Arm A, 3 (25%) in Arm B, and 1 (17%) in Arm C. The RP2D for Arm A was ipatasertib 300 mg daily, carboplatin AUC2 and paclitaxel 80 mg m^−2^ (days 1, 8, and 15 every 28 days). The RP2D for Arm B was ipatasertib 400 mg daily/carboplatin AUC2 (days 1, 8, and 15 every 28 days). The likely RP2D for Arm C was ipatasertib 300 mg (21 days on, 7 days off), capecitabine 750 mg m^−2^ bid (7 days on 7 days off), and atezolizumab 840 mg days 1 and 15 every 28 days, with 1 DLT in 6 patients (the study stopped early prior to possible dose escalation).

### Response and Survival

The best responses for patients in Arm A (carboplatin/paclitaxel plus ipatasertib) included 2 (20%) PR (for the RP2D it was 2/7, 29%), 4 (40%) SD, and 4 (40%) PD. Arm B (carboplatin plus ipatasertib) best response included 1 (8%) CR, 2 (17%) PR, 6 (50%) SD, and 3 (25%) PD. Arm C (capecitabine, atezolizumab, and plus ipatasertib) best response included 2 (33%) PR, 3 (50%) SD, and 1 (17%) was called progressive disease, but we noted the patient was HER2+ on biopsy of progressive disease (Table 4). The overall response rate (ORR = CR + PR) was 20% for Arm A, 25% for Arm B, and 33% for Arm C. Taken altogether (*N* = 28), the overall responses were: 1 (4%) CR, 6 (21%) PR, 13 (46%) SD, 7 (25%) PD, and 1 (4%) HER2+ at time of progression. A total of 10 patients had clinical benefit rate (CBR = CR + PR + SD) at 6 months without progression, including 2 (20%) in Arm A, 5 (42%) in Arm B, and 3 (50%) in Arm C. Duration of response (CR and PR) was 4 and 7 months for Arm A; 6, 9, and 15 months for Arm B; and 9 and 15 months for Arm C (1 patient was still on treatment). The median follow-up for Arm A was 24 months and Arm C was 20 months. Reverse Kaplan–Meier method could not be used to determine median follow-up for arm B; but was 10 months and 21 months for the 2 patients on follow-up.

**Table 4.** Response per RECIST 1.1 (*N* = 28).

Best response	Overall, *N* = 28	Arm A, *N* = 10	Arm B, *N* = 12	Arm C, *N* = 6
CR	1 (4%)	0 (0%)	1 (8%)	0 (0%)
PR	6 (21%)	2 (20%)^a^	2 (17%)	2 (33%)
SD	13 (46%)	4 (40%)	6 (50%)	3 (50%)
PD	8 (29%)	4 (40%)	3 (25%)	1 (17%)^b^
ORR (CR + PR)	7 (25%)	2 (20%)	3 (25%)	2/6 (33%)^c^
CBR (CR + PR + SD) at 6 months	10 (36%)	2 (20%)	5 (42%)	3 (50%)

^a^Both at RP2D, resulting in 2/7 or (29%) at RP2D.

^b^Patient was HER2+ at progression.

^c^Excluding HER2+ patient at PD in Arm C, ORR is 2/5 (40%).

**Figure 2.**
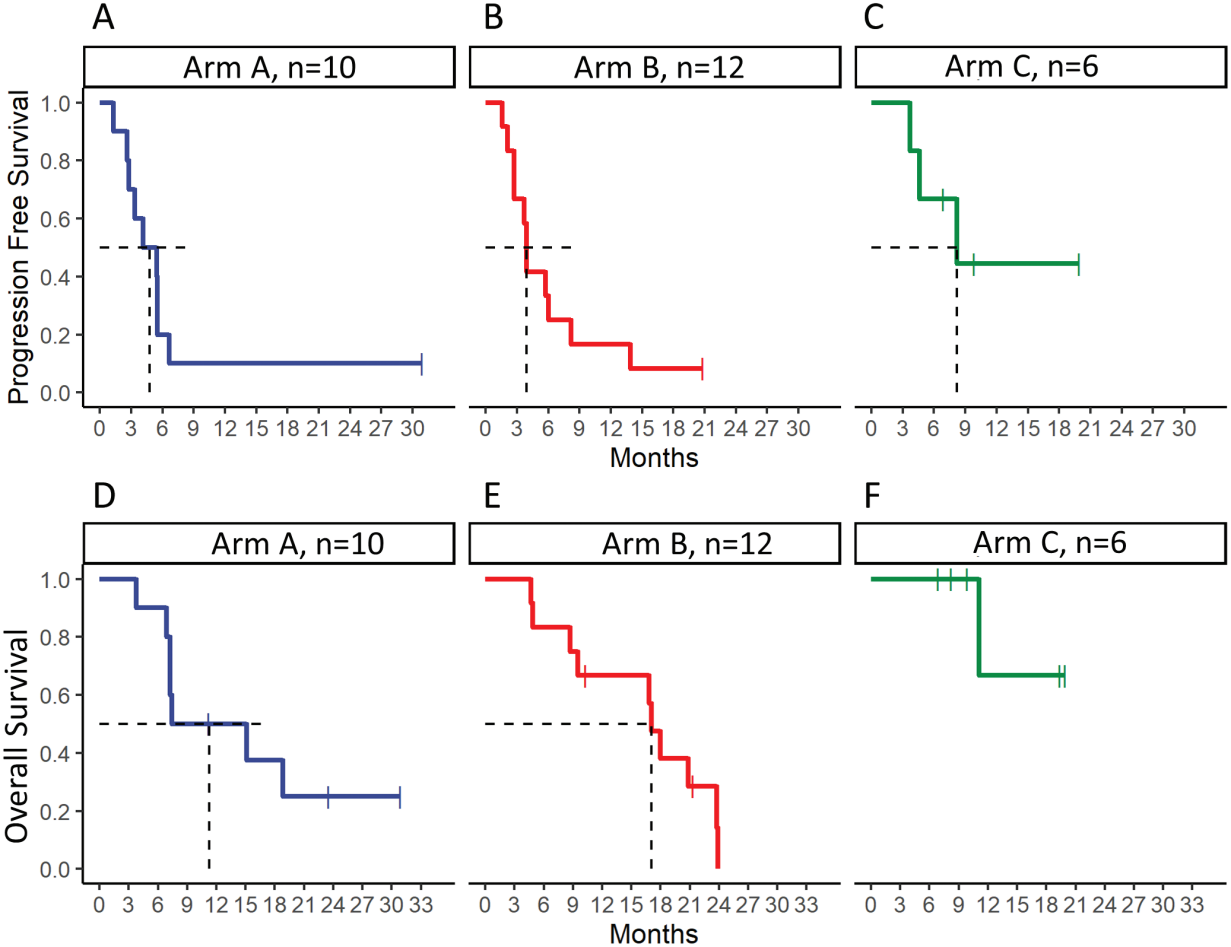
Progression-free survival (PFS) and overall survival (OS) (*N* = 28). (**A**) Arm A (*N* = 10) median PFS was 4.8 months (95% CI 2.8, NA); (**B**) Arm B (*N* = 12) median PFS was 3.9 months (95% CI 2.8, NA); (**C**) Arm C (*N* = 6) median PFS was 8.2 months (95% CI 4.6, NA); (**D**) Arm A (*N* = 10) median OS was 11.2 months (95% CI 7.2, NA); (**E**) Arm B (*N* = 12) median OS was 17.0 months (95% CI 9.5, NA); and (**F**) Arm C (*N* = 6) median OS was NA (95% CI 11.1, NA). PFS, progression-free survival; OS, overall survival; NR, not reached.

The median PFS for Arm A was 4.8 months (95% CI 2.8, NA), Arm B was 3.9 months (95% CI 2.8, NA), and Arm C was 8.2 months (95% CI 4.6, NA). The median OS for Arm A (*N* = 10) was 11.2 months (95% CI 7.2, NA), Arm B (*N* = 12) was 17.0 months (95% CI 9.5, NA), and Arm C (*N* = 6) is NA (95% CI 11.1, NA; Fig. 2).

**Figure 3.**
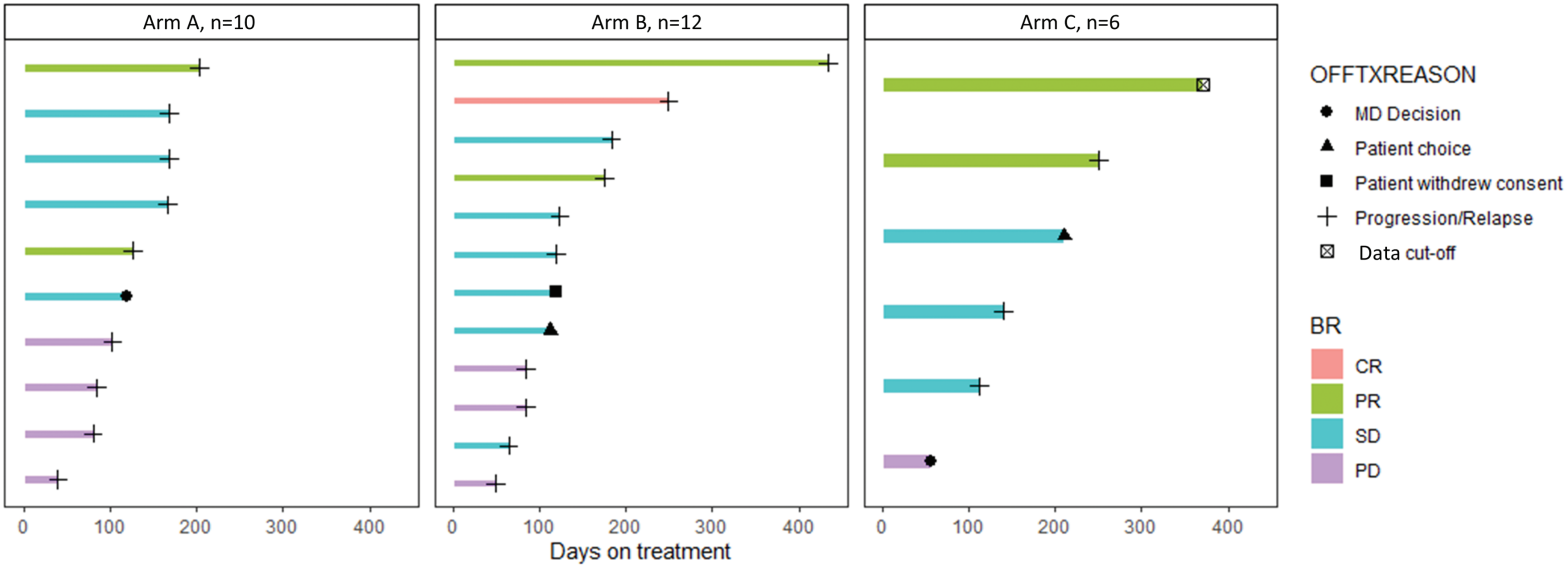
Swimmer plot (*N* = 28). Response to treatment over time is shown for Arm A (*n* = 10), Arm B (*n* = 12), and Arm C (*n* = 6), including best response and reason for patient going off treatment.

A swimmer plot with response to treatment over time is shown for Arm A (*n* = 10), Arm B (*n* = 12), and Arm C (*n* = 6), including best response and reason for patient going off treatment (Fig. 3). One patient on Arm C went off the study as of June 2022 without progression.

### Immune Correlatives

PD-L1 testing for Arm C patients showed 4 patients who were PD-L1 positive by SP142 and 2 patients who were PD-L1 negative (Supplemental Table S1). No clear association was identified between PD-L1 or stromal TILs with best response.

### Androgen Receptor

A total of 10 patients were AR positive (defined by IHC AR ≥2+ and ≥30%, *n* = 10) and 17 were AR negative (1 had no AR results). In the AR positive population, the responses were 1/10 CR, 3/10 PR, 5/10 SD, and 1/10 PD or 4/10 responders (40%; Supplemental Table S1). AR negative patients included 3/17 PR, 8/17 SD, and 6/17 PD or 3/17 responders (18%). The difference was not significant (*P* = .2, 1–sided Fisher’s Exact test).

### Tumor Genomics

NextGen exome sequencing was performed with commercial testing (Supplemental Table S2). A total of 15 patients had PIK3CA/AKT/PTEN and 12 had no alterations (1 had no genomic results). PIK3CA alterations were present in 6/15 (40%) of patients, PIK3CD in 1/15 (7%), PIK3R1 in 2/15 (13%), AKT1 in 1/15 (7%), PTEN loss in 3/15 (20%), and PIK3CA plus PTEN loss in 2/15 (13%). Among these, responses were 1/15 CR, 4/15 PR, 7/15 SD, and 3/15 PD or 5/15 (33%) responders. Patients with no PIK3CA/AKT/PTEN alterations included 2/12 PR, 5/12 SD, and 5/12 PD or 2/12 (17%) responders. ORR for patients with PIK3CA/AKT/PTEN alterations was numerically higher but not statistically significant (*P* = .30, 1–sided Fisher’s Exact test).

## Discussion

The results of the current trial provide evidence that combining ipatasertib with continuous daily dosing with weekly carboplatin or carboplatin/paclitaxel is safe and has modest clinical activity in patients with mTNBC. In addition, ipatasertib (21 days on and 7 days off) combined with capecitabine and atezolizumab is safe and showed clinical activity. In patients with PIK3CA/AKT/PTEN alteration, ORR was 33% (5/15, including 1 CR). In AR+ TNBC, ORR was 40% (4/10). Due to limited sample size, these results are hypothesis-generating. The KEYNOTE-355 trial already established ICI plus chemotherapy for standard management of first–line patients with PD-L1+ TNBC.^6^ The data presented by this study may provide proof of principle for a combination of ipatasertib, capecitabine, and atezolizumab for treatment of mTNBC.

ICI plus chemotherapy combinations are now standard of care for PD-L1 positive mTNBC. Atezolizumab was initially granted FDA approval in March 2019 based on significant PFS benefit when atezolizumab was combined with nab–paclitaxel vs nab–paclitaxel alone seen in from IMpassion130 trial (HR, 0.60; 95% CI, 0.48-0.77; *P* < .0001).^4^ The Phase III confirmatory trial IMpassion131 trial did not meet the primary end point of PFS benefit as in PD-L1+ mTNBC as first–line therapy (HR, 0.82; 95% CI, 0.60-1.12; *P* = .20) and no OS benefit seen in the intention to treat population. In the KEYNOTE 355 study, patients with PD-L1+ TNBC (22C3 antibody, CPS ≥ 10) pembrolizumab plus chemotherapy had improved PFS compared with chemotherapy alone (9.7 vs 5.6 months; HR 0.65, 95% CI: 0.49-0.86).^6,37^ The precise underlying etiology of efficacy difference observed in these ICIs remains unclear. Capecitabine was previously tested in combination with ICIs. Shah et al. reported an ORR of 26% and CBR of 28% (*N* = 15) in a cohort of patients with mTNBC treated with capecitabine and pembrolizumab combination.^28^ Page et al. reported capecitabine and pembrolizumab demonstrated a 12-week ORR of 43% and PFS of 5.6 months.^38^ In our study, ORR of 40% with ipatasertib, capecitabine, and atezolizumab in PD-L1 unselected patients is significant, despite the limited number of patients.

In LOTUS trial, ipatasertib added to first–line paclitaxel for mTNBC improved PFS with an enhanced effect in patients with PIK3CA/AKT1/PTEN–altered tumors.^15^ Final OS results show a numerical trend favoring ipatasertib–paclitaxel (median OS 23.1 vs 18.4 months in ipatasertib vs placebo arm, stratified HR 0.62 [95% CI, 0.37-1.05]).^33^ However, the Phase III IPATunity130 cohort A failed to show PFS benefit from the addition of ipatasertib to paclitaxel in patients with mTNBC harboring PIK3CA/AKT1/PTEN mutations.^39^ Similarly, adding ipatasertib to paclitaxel did not improve efficacy in PIK3CA/AKT1/PTEN–altered HR+ HER2–negative advanced breast cancer.^17^ In the FAIRLANE trial, adding ipatasertib to 12 weeks of paclitaxel for early TNBC did not clinically or statistically significantly increase the pCR rate, although the ORR by MRI was numerically higher with ipatasertib.^40^ In the current study, the modest efficacy seen in the total population is consistent with IPATunity130 cohort A. Preclinical studies have shown co–administration of ipatasertib and ICI may enhance efficacy by retaining the stem–like phenotype of memory T cells, thus preventing exhaustion.^41,42^ A multi–arm basket-trial with immunotherapy–based treatment is currently ongoing (MORPHEUS TNBC NCT03424005) to further test ipatasertib combinations.^43^

TNBC is molecularly heterogeneous with at least 4-6 molecular subtypes defined by mRNA expression. Among these, approximately 10% are luminal androgen receptor positive (LAR).^44,45^ Lehmann et al. demonstrated that activating PIK3CA mutations is enriched in LAR TNBC; the growth and viability of androgen receptor (AR) positive TNBC cell line models are significantly reduced after treatment with PI3K inhibitors used in combination with an AR antagonist.^46^ LAR TNBC PDX models were significantly enriched in *PIK3CA* and *AKT1* mutations, had higher levels of luminal–androgen–like gene expression, and had a higher PI3K pathway protein activation score than other TNBC subtypes.^47^ These results provide rationale for AR antagonists in combination with PI3K/AKT/mTOR inhibitors. In the current study, our finding of ORR 44% in AR+ TNBC is encouraging. Confirmatory studies are required to better understand the role of ipatasertib in AR+ TNBC.

Other AKT inhibitors are currently undergoing vigorous clinical investigation. In the PAKT trial, the addition of the pan-AKT inhibitor capivasertib to first-line paclitaxel therapy for TNBC resulted in longer PFS and OS. The median PFS was 5.9 months with capivasertib plus paclitaxel and 4.2 months with placebo plus paclitaxel (hazard ratio [HR], 0.74; 95% CI, 0.50-1.08; 1-sided *P* = .06). Median OS was 19.1 months with capivasertib plus paclitaxel and 12.6 months with placebo plus paclitaxel (HR, 0.61; 95% CI, 0.37-0.99; 2-sided *P* = .04). In patients with *PIK3CA*/*AKT1*/*PTEN*–altered tumors (*n* = 28), median PFS was 9.3 months with capivasertib plus paclitaxel and 3.7 months with placebo plus paclitaxel (HR, 0.30; 95% CI, 0.11-0.79; 2-sided *P* = .01).^48^ The results of the Phase III randomized study with capivasertib and CAPItello-290 are eagerly awaited (NCT03997123).

There are several limitations of this study including early discontinuation due to withdrawal of funding, limited enrollment per arm, variation of chemotherapy backbone, lack of randomized Phase II design, and underlying molecular heterogeneity of mTNBC. An appropriately designed randomized Phase II trial may facilitate better understanding the role of ipatasertib in treating patients with TNBC who have specific molecular alterations or phenotypes.

## Conclusions

Results from this study showed that continuous dosing of ipatasertib in combination with carboplatin-based therapy or capecitabine/atezolizumab is safe; however, moderate efficacy was seen in TNBC patients with PI3K/AKT/PTEN alterations. Encouraging efficacy was seen in luminal androgen receptor TNBC. Future studies with larger patient cohorts and randomized designs are required to confirm the current findings.

## Supplementary Material

oyad026_suppl_Supplementary_Tables

## Data Availability

The data underlying this article will be shared on reasonable request to the corresponding author.
