# Supplementary material for: Phase I Trial of Ipatasertib Plus Carboplatin, Carboplatin/Paclitaxel, or Capecitabine and Atezolizumab in Metastatic Triple-Negative Breast Cancer
Source: Oncologist. 2023 Apr 6;28(7):e498–507. doi: 10.1093/oncolo/oyad026 (PMC10322142; doi:10.1093/oncolo/oyad026)
Supplement: oyad026_suppl_Supplementary_Tables [file oyad026_suppl_supplementary_tables.docx]

### Supplemental Table 1. Tumor characteristics. Pre-treatment tumor biopsies were assessed for PD-L1 expression, tumor infiltrating lymphocytes, and AR expression.

| Arm | Best Response | PD-L1 (SP142) | TILs (%) | AR (%) | AR > 2+ and >30%^1^ |
| --- | --- | --- | --- | --- | --- |
| A | PR | NEG | 3% | 95% at 2-3+ | Pos |
| A | PR | NA | 5% | 0% | Neg |
| A | SD | NA | 2% | 80% at 2+ | Pos |
| A | SD | POS | 10% | <1% at 2+ | Neg |
| A | SD | NA | 10% | 0% | Neg |
| A | SD | NA | 5% | 0% | Neg |
| A | PD | NA | 2% | 70% at 1+ | Neg |
| A | PD | POS | 20% | 1-5% at 1+ | Neg |
| A | PD | NEG | 10% | 0% | Neg |
| A | PD | NA | 2% | NA | NA |
| B | CR | NA | 5% | 60% at 2+ | Pos |
| B | PR | NEG | 2% | 1-10% at 1-2+ | Neg |
| B | PR | NA | 2% | 5% at 1+ | Neg |
| B | SD | NA | 10% | 60% at 2+ | Pos |
| B | SD | POS | 10% | 30% at 2+ | Pos |
| B | SD | NA | 10% | 20% at 1+ | Neg |
| B | SD | NA | 2% | 20% at 1+ | Neg |
| B | SD | NEG | 2% | 10-20% at 1-2+ | Neg |
| B | SD | POS | 40% | 0% | Neg |
| B | PD | NA | 10% | 30% at 1-2+ | Pos |
| B | PD | NEG | 3% | 30% at 1+ | Neg |
| B | PD | NA | 5% | 0% | Neg |
| C | PR | NEG | 3% | 100% at 3+ | Pos |
| C | PR | POS | 10% | 90% at 2-3+ | Pos |
| C | SD | POS | NA | 75% at 2+ | Pos |
| C | SD | POS | NA | 40% at 1-2+ | Pos |
| C | SD | NEG | NA | 0% | Neg |
| C | PD | POS | 15% | 1-10% at 1-2+ | Neg |

^1^AR defined by IHC AR ≥2+ and ≥30%.

Abbreviations: CR, complete response; PR, partial response; SD, stable disease; PD, progression of disease; NA, not applicable; TILs: tumor infiltrating lymphocytes; AR, androgen receptor**.**

### Supplemental Table 2. Genomic alterations. Pre-treatment tumor biopsies were assessed for genomic alterations and tumor mutation burden.

| Arm | Best Response | Tissue Seq | Platform | TMB (m/MB) | Gene alteration | PIK3CA/AKT/PTEN Pathway alteration |
| --- | --- | --- | --- | --- | --- | --- |
| A | PR | Liver | Tempus | 2.1 | GPS2, PIK3CA, MLL3 | Pos |
| A | PR | Lung | GEM | 2 | ATRX, CCND1, FGF3, FGF4, FGF19, NOTCH2,TP53, DMAP1/NOTCH2 | Neg |
| A | SD | Blood | Guardant | NA | No alterations | Neg |
| A | SD | LN | Tempus | 8.9 | ARID1B, BRCA1, BRCA1, TP53 | Neg |
| A | SD | Lung | Foundation | 16 | BCOR, CDKN2A, NRAS, PIK3R1, TP53, XRCC2 | Pos |
| A | SD | Chest wall | Foundation | 0 | CREBBP, MYC, TP53 | Neg |
| A | PD | Breast | Tempus | 2.1 | BRCA1, BRCA1, TP53 | Neg |
| A | PD | LN | Tempus | 2.1 | CCND3, CCNE1, PIK3R1, PHGDH, TFEB, TP53 | Pos |
| A | PD | Breast | Tempus | 1.1 | CDKN2A, CDKN2B, MLL3, MTAP, TP53 | Neg |
| A | PD | Skin | GEM | 2 | EGFR, MYC, PIK3CA, TERT, TP53, ZHX1/CHMP4C | Pos |
| B | CR | Chest wall | HopeSeq | Cannot be determined | AKT1, NF1, TP53 | Pos |
| B | PR | Chest wall | Tempus | 3.7 | PHGDH, PTEN, MYC, RECQL4, TP53 | Pos |
| B | PR | Lung | Tempus | 8.4 | NOTCH1, PIK3CA, TP53, NFIX-MAST1 | Pos |
| B | SD | Breast | Tempus | 9.5 | FAS, PTEN, RB1, TP53, ADAMTS15, MRPL18/FOXP1 | Pos |
| B | SD | Skin | Caris | 6 | PIK3CA, TP53 | Pos |
| B | SD | Lung | GEM | 4 | ASXL1, ATRX, BRCA1, CDKN2B, TP53 | Neg |
| B | SD | NA | NA | NA | Not done | NA |
| B | SD | Soft tissue | Tempus | 7.9 | NF1, PIK3CA, SDHA, SPEN, TP53 | Pos |
| B | SD | Supraclavicular node | Tempus | 5.3 | BRCA2, BRD4, CDKN2A, CDKN2B, CHEK2, CHEK2, KMT2D, MTAP, NBN, TP53 | Neg |
| B | PD | Blood | GEM | 2 | CCNE1 | Neg |
| B | PD | Liver | Tempus | 1.6 | BAP1, BCL2L1, HRAS, KAT6A, PBRM1, TP53 | Neg |
| B | PD | Breast | Foundation | 8 | MYC, TP53 | Neg |
| C | PR | Breast | Tempus | 3.2 | CDH1, NCOR1, MAP2K4, PIK3CA, PTEN, TP53 | Pos |
| C | PR | Liver | Tempus | 0.5 | No alterations | Neg |
| C | SD | Breast | Tempus | 1.6 | CKS1B, GATA3, MSH2, PHGDH, PTEN, RECQL4, TERT, TP53, SEC22B-NOTCH2 | Pos |
| C | SD | Triceps | Tempus | 1.1 | PIK3CA, PTEN, TP53 | Pos |
| C | SD | Breast | Tempus | 7.4 | CDKN2A, CDKN2B, PIK3CD, PPM1D, PTPRD, RPS6KB1, TP53, RPS6KB1-VMP1 | Pos |
| C | PD | Soft tissue | Tempus | 10 | PIK3CA, TET2, TP53, LRRC37A11P-RARA | Pos |

Abbreviations: CR, complete response; PR, partial response; SD, stable disease; PD, progression of disease; NA, not applicable; TMB, tumor mutation burden.
